# Differential Expression of IL-17, 22 and 23 in the Progression of Colorectal Cancer in Patients with K-ras Mutation: Ras Signal Inhibition and Crosstalk with GM-CSF and IFN-γ

**DOI:** 10.1371/journal.pone.0073616

**Published:** 2013-09-06

**Authors:** Savvas Petanidis, Doxakis Anestakis, Maria Argyraki, Margarita Hadzopoulou-Cladaras, Athanasios Salifoglou

**Affiliations:** 1 Department of Chemical Engineering, Aristotle University of Thessaloniki, Thessaloniki, Greece; 2 Laboratory of General Biology, Medical School, Aristotle University of Thessaloniki, Thessaloniki, Greece; 3 Department of Genetics, Development and Molecular Biology, School of Biology, Aristotle University of Thessaloniki, Thessaloniki, Greece; The University of Hong Kong, China

## Abstract

Recent studies have suggested that aberrant K-ras signaling is responsible for triggering immunological responses and inflammation-driven tumorigenesis. Interleukins IL-17, IL-22, and IL-23 have been reported in various types of malignancies, but the exact mechanistic role of these molecules remains to be elucidated. Given the role of K-ras and the involvement of interleukins in colorectal tumorigenesis, research efforts are reported for the first time, showing that differentially expressed interleukin IL-17, IL-22, and IL-23 levels are associated with K-ras in a stage-specific fashion along colorectal cancer progression. Specifically, a) the effect of K-ras signaling was investigated in the overall expression of interleukins in patients with colorectal cancer and healthy controls, and b) an association was established between mutant K-ras and cytokines GM-CSF and IFN-γ. The results indicate that specific interleukins are differentially expressed in K-ras positive patients and the use of K-ras inhibitor Manumycin A decreases both interleukin levels and apoptosis in Caco-2 cells by inhibiting cell viability. Finally, inflammation-driven GM-CSF and IFN-γ levels are modulated through interleukin expression in tumor patients, with interleukin expression in the intestinal lumen and cancerous tissue mediated by aberrant K-ras signaling. Collectively, the findings a) indicate that interleukin expression is influenced by ras signaling and specific interleukins play an oncogenic promoter role in colorectal cancer, highlighting the molecular link between inflammation and tumorigenesis, and b) accentuate the interwoven molecular correlations as leads to new therapeutic approaches in the future.

## Introduction

Colorectal cancer is the second most prevalent form of cancer worldwide. Currently, in most of the developing countries, there are no organized screening and diagnostic programs [1], [2]. Previous studies have shown that colorectal cancer is a multifactorial disease, in which the expression of many specific genes, known as oncogenes or tumor suppressors, is abnormally altered [3]. In this regard, the PIK3CA gene, which is involved in the PI3K/AKT signaling pathway, is up-regulated in colorectal cancer. The tumor suppressor gene phosphatase and tensin homolog (PTEN) is down-regulated due to a genetic mutation or deletion [4]. However, molecular mechanisms of colorectal carcinogenesis remain to be elucidated. Toward such efforts, it is crucial to identify specific molecular markers for the detection and identification of mechanisms contributing to colorectal carcinogenesis. One such representative biomarker is K-ras, an oncogene with guanosine triphosphate (GTP) binding properties [5]. Due to its ability to interact with key signaling molecules including the signal transducer and activator of transcription (STAT), phosphoinositide 3-kinase (PI3K), and mitogen-activated protein kinase (MAPK), the K-ras gene delivers an essential function in cell division, cell growth and differentiation. Thus, mutations in the K-ras gene (especially, single nucleotide substitutions) are implicated in most types of tumors, including lung adenocarcinoma, lung cancer, ductal carcinoma of the pancreas, and colorectal carcinoma [6].

Over the past few years, evidence has demonstrated that interleukins carry out important functions in tumor development, cell differentiation, inflammation and metastasis [7], [8]. In this respect, IL-17, which is largely produced by activated memory T lymphocytes, stimulates both innate immunity and host defense, and plays an active role in inflammatory diseases, autoimmune diseases, and cancer. More specifically, IL-17 induces the expression of nuclear factor-kappa B (NF-κB), chemokines CXCL8, CXCL6 and CXCL1, growth factors G-CSF, GM-CSF (granulocyte-macrophage colony-stimulating factor), IL-6, and adhesion molecules (ICAM-1), leading to augmented neutrophil accumulation, granulopoeisis, and inflammatory responses [9], [10]. On the other hand, IL-22 acts as a mediator of cellular inflammatory responses by activating intracellular kinases (JAK1, Tyk2, and MAP kinases) and transcription factors such as STAT3 [11]. Furthermore, IL-22 exhibits anti-apoptotic and tumorigenic functions, with recent data showing that over-expression of that molecule protects lung cancer cell lines from apoptosis via a) activation of STAT3 and its downstream anti-apoptotic proteins Bcl-2 and Bcl-xL, and b) inactivation of extracellular signal-regulated kinases [12]. Likewise, IL-23 plays a key role in chronic intestinal inflammation and its up-regulation in malignant tissues parallels augmented levels of the “metastatic biomarker” matrix metalloproteinase MMP-9, tumor necrosis factor TNF-alpha, and increased levels of angiogenesis [13], [14], [15].

In an effort to discover molecular links between tumorigenic and immuno-inflammation pathways in cancer physiology, research was launched in our labs to probe into the interactions and potential interwoven roles that the aforementioned molecular targets might play in colorectal multistage cancer progression. To this end, we report herein for the first time that a) the specific interleukins are up-regulated in colorectal carcinoma compared to healthy colorectal tissues, b) interleukins are over expressed in all K-ras patients and can promote cell growth and inhibit cell apoptosis in Caco-2 colorectal cancer cells through the ras signaling pathway, c) use of the K-ras inhibitor Manumycin A decreases interleukin levels, and decreases apoptosis in Caco-2 colorectal cancer cells by inhibiting cell viability, and d) GM-CSF and IFN-γ (interferon gamma) are modulated through interleukin expression either positively or negatively. The collective observations shed light onto the functional association between interleukins in inflammation, ras-signaling and colorectal tumorigenesis, thereby potentially aiding in the development of future cancer detection and therapeutics.

## Materials and Methods

### Ethics Statement

This study was performed with the approval of the Institutional Review Board of the AHEPA Hospital, Medical School, Aristotle University of Thessaloniki. The paper satisfies PLOS ONE policies regarding human subject research. A written informed consent was received from every patient. Written consents were obtained, prior to surgery, from patients voluntarily involved in the usage of tissues solely for research purposes. Patients had read and understood the patient information document provided, and the aims and methods of this study had been fully explained to them. Patients involved had given written informed consent to authors of this manuscript for publication of these data. The clinical investigation was conducted according to the guidelines expressed in the Declaration of Helsinki.

### Reagents

Manumycin A was purchased from Sigma (Sigma Germany, Europe). DMEM-Hepes, PBS (phosphate buffer saline), FBS (fetal bovine serum), and Trypsin-EDTA reagents were purchased from Invitrogen (Invitrogen, Darmstadt, Germany). DMSO (Dimethyl sulfoxide) and Tris-EDTA (Tris-Ethylene Diamine Tetraacetic Acid) were purchased from Applichem (Applichem, Darmstadt, Germany). The K-ras (A-18) goat polyclonal IgG-horseradish peroxidase (HRP), mouse anti-goat IgG-HRP, and GAPDH(L-20) goat polyclonal IgG-HRP antibodies for Western blot analysis were obtained from Santa Cruz (Santa Cruz, California, USA). The human IL-17, IL-22, IL-23, GM-CSF and IFN-γ were detected using anti-human monoclonal antibodies from Santa Cruz. For the preparation of aqueous stock solutions (1.0 Μ), Manumycin A was dissolved in 10 mM Tris, pH 7.4. The Manumycin A stock solution was filtered through a 0.22 µm syringe filter and subsequently aliquoted and stored in the dark at room temperature. Tris-EDTA (TE) buffer was prepared from a 1 M Tris, pH 8, and a 100 mM EDTA stock solution. The final concentrations were 50 mM Tris and 1 mM EDTA. The so prepared stock was filtered through a 0.22 µm filter, then aliquoted and stored in the dark at room temperature.

### Patients

A total of 92 colorectal cancer (CRC) patients, 37–88 years of age, were from the AHEPA Hospital, Medical School, Aristotle University of Thessaloniki (Table S1). Of these patients, 28 harbored K-ras mutations (24 with G12V and 4 with G12D). These patients underwent resection of colorectal cancer (stages B1–D) between 2009 and 2012. In addition, 56 healthy volunteers who a) met the requirements of having matched gender and similar age with the samples of CRC patients, and b) exhibited no colonic adenomas, were selected as controls. Patients with inflammatory conditions, including infectious or collagen diseases, were excluded. All patients were classified according to UICC stage classifications using resected specimens. Blood samples were obtained by venipuncture before colorectal surgery. Each sample was centrifuged at 3,000 g for 5 min, and then frozen at −80°C until analysis.

### DNA and RNA Extraction

Formalin-fixed, paraffin-embedded patient tumor sections were selected by a pathologist to confirm the diagnosis and define tumor-enriched areas for dissection. The isolated cancer cells were lysed in buffer (0.2 M tris HCl, 0.5 M NaCl, 0.01 M EDTA, 1% SDS, 1 M sodium acetate) containing Proteinase K at 60°C for 72 hr and DNA extraction was performed with the use of the Qiagen DNA and RNA Purification Kit according to manufacturer instructions (Qiagen, Athens, Greece). For RNA extraction, cancer cells were re-suspended in 400 µl RNA lysis buffer (0.5 M EDTA, 10% SDS, 1 M Tris pH 7.5) supplemented with 300 mg proteinase K and incubated at 60°C for 16 hr until the tissue had been completely solubilized. RNA was purified by Trizol reagent (Invitrogen, Paisley, UK), subsequently treated with DNase to avoid genomic DNA contamination, and stored at −80°C until use.

### Enzyme-linked Immunosorbent Assays (ELISA)

Prior to assaying, frozen tissue samples were brought to room temperature. Subsequently, they were washed in an isotonic solution of sodium chloride (Sigma), cut into pieces, weighed and homogenized at 4°C in lysis buffer (50 mM HEPES pH 7.4, 5 mM CHAPS, 5 mM 1 DTT). Sample processing took place in a mass scale of 1 g tissue/10 ml lysis buffer. The homogenized tissues remained at −20°C for 24 hr and then centrifuged at 20,000 g for 15 min at 4°C. Supernatants (10 µl per well) were used for quantitative stage-specific (B1, B2, C1, C2, D) detection of IL-17, IL-22, and IL-23 through enzyme-linked immunosorbent assays from Cytoscreen (BioSource International, Camarillo, CA). The detection limits for the ELISA kits, as specified by the manufacturer, were 9 pg/ml (IL-17), 8 pg/ml (IL-22) and 4 pg/ml (IL-23). Manumycin A treatment was applied to all stage D patient samples for all three interleukins investigated.

### Reverse Transcriptase Polymerase Chain Reaction (RT-PCR)

RT-PCR was performed on patient tissue samples for IL-17, IL-22, IL-23, GM-CSF and IFN-γ. GAPDH (glyceraldehyde 3-phosphate dehydrogenase) was used as an internal control. Amplification was performed in a total volume of 20 µl per reaction, containing 10 µl of PCR Master Mix (Applied Biosystems, Warrington, UK), 5 µl of primers (1 pmol/µl), and 5 µl of cDNA (40 ng RNA). RT-PCR primers for IL-17, 1L-22, IL-23, GM-CSF, IFN-γ and GAPDH are listed in Table S2. The PCR reaction parameters were set as follows: 95°C for 15 minutes followed by 40 cycles of PCR reacting at 94°C for 15 seconds and 60°C for 1 minute. Melting curve analysis of amplification products was performed at the end of each PCR reaction by increasing the temperature from 60 to 95°C with a temperature transition rate of 0.1°C per second, allowing us to distinguish genuine products from non-specific products and primer dimers. PCR products were also separated on a 1.5% agarose gel to visualize the formation of correct PCR products. The relative quantification (RQ) of gene expression was calculated assuming 100% efficient PCR, in which each CT value of the reactions was normalized to GAPDH mRNA expression.

### K-ras Mutation Detection and Sequence Analysis of K-ras G12 Codon

All patient tumor tissue specimens were snap-frozen and preserved in liquid nitrogen before use. Paraffin sections were then prepared and one section from each case was chosen for Hematoxylin-Eosin staining. An experienced pathologist analyzed the Hematoxylin-Eosin section to confirm the presence of tumor tissue. Subsequently, tissue samples from at least five serial sections were macrodissected to ensure that the specimens contained at least 80% tumor cells. DNA was isolated from these tissue samples using the Qiagen DNA extraction Kit (Qiagen, Berlin, Germany) according to manufacturer instructions. Subsequently, mutations in codon 12 of K-ras exon 1 were detected in these genomic DNA samples by PCR-based direct sequencing. The PCR primers for K-ras used were K-ras-Forward: 5′-AAGGCCTGCTGAAAGTGACTG-3′ and K-ras-Reverse: 5′-CTGGTGCAGGACCATTCTTCAG-3′. The PCR reaction was carried out in a total volume of 50 µl containing 150 ng of the extracted genomic DNA. PCR amplification conditions were as follows: initial denaturation at 94°C for 1 min, followed by 40 cycles of denaturation at 94°C for 30 s, annealing at 55°C for 30 s, and extension at 72°C for 30 s, with a final extension step at 72°C for 5 min in a TP-600 thermal cycler (TaKaRa). PCR products were then analyzed using 2.5% agarose gel electrophoresis and purified for further direct DNA sequencing through an ABI-3730XL DNA Analyzer (Applied Biosystems, Europe).

### Immunohistochemical Analysis of IL-17, IL-22, and IL-23

Paraffined sections of human colon biopsies from patients with colorectal cancer were treated with anti-human IL-17, anti-human IL-22, and anti-human IL-23 monoclonal antibodies (mAb) (Santa Cruz Biotechnology, Santa Cruz, CA). Staining with mouse IgG1 isotype was used as the negative control. Images were obtained through a Carl Zeiss microscope using image analysis software (Carl Zeiss, Berlin, Germany).

### Western Blot Analysis of IL-17, IL-22, and IL-23

Collected colorectal patient tissue samples were washed twice with ice-cold PBS and lysed in 100 µL of ice-cold lysis buffer per 1×10^6^ cells. The lysates were incubated on ice for 10 min, vortexed for 45 s and maintained on ice for another 10 min. Following centrifugation at 14,000 g and 4°C for a period of 15 min, the supernatant was collected and proteins were quantified by the Bradford method. Lysate proteins dissolved in 6xLaemmli sample buffer were separated (30 µg/lane) using SDS–polyacrylamide gel electrophoresis (10% acrylamide) and electro-transferred to PVDF (polyvinylidene difluoride) membranes. After blocking with 5% non-fat milk in TBST buffer (50 mM Tris, 150 mM NaCl, 0.05% Tween 20, pH 7.6), the membranes were incubated for 90 min with the appropriate dilution of the primary antibody in TBST plus 1% non-fat milk. After washing, the membranes were incubated with the appropriate dilution of the horse radish peroxidase-conjugated secondary antibody in TBST plus 1% non-fat milk. Finally, the blots were developed by ECL (enhanced chemiluminescence) and digitized through a BioSpectrum 500 Imaging System. GAPDH was used as an internal control in all experiments.

### ELISA SPOT for GM-CSF and IFN-γ

IFN-γ-producing cells from patient blood samples were quantitated with an IFN-γ ELISPOT kit (Diaclone, Besancon, France) according to manufacturer instructions, with minor modifications. Membranes were coated with human IFN-γ antibodies and incubated overnight at 37°C. Then, 1×10^5^ PBMCs (peripheral blood mononuclear cells) from colorectal cancer patients were added onto the plates and incubated for 26 hr at 37°C with 5% CO_2_. Cells were removed and the biotinylated antibodies against human IFN-γ were added onto the membranes. Spots were counted by an automated plate counter from Applied Biosystems (Applied Biosystems, Europe).

GM-CSF producing cells from PBMCs of patients were quantitated by using a GM-CSF ELISPOT kit (R&D Biosystems, Minneapolis, USA) according to manufacturer instructions. Briefly, 96-well filtration ELISPOT plates were coated with 4 µg/ml of GM-CSF antibody in 100 µl of reagent diluent overnight at 37°C. Plates were washed twice with wash buffer (PBS with 0.5% Tween 20) and blocked for 2 hr at room temperature in blocking buffer containing PBS, supplemented with 1% BSA and 5% Sucrose. ELISPOT plates were incubated at 37°C, 5% CO_2_, and 100% humidity. Wells containing cells only served as negative control. Spots were enumerated by an automated plate counter (Applied Biosystems).

### Cell Culture

Human epithelial colorectal adenocarcinoma Caco-2 cells were obtained from the American Type Culture Collection (ATCC, Manassas, VA, USA) and were grown in DMEM (dulbecco’s modified eagle medium) supplemented with 10% fetal bovine serum, 2 mM L-glutamine, 1 mM sodium pyruvate, 100 U/ml penicillin, and 100 µg/ml streptomycin (Biochrom, Germany). The cell cultures were grown to confluence and maintained in a humidified atmosphere at 37°C and 5% CO_2_. The Caco-2 cells were incubated with fetal bovine serum-free medium for 24 hr prior to the treatment with Manumycin A at a final concentration in the range 50–300 µM. Three independent sets of experiments were performed on a Manumycin A treatment.

### MTT Cell Viability Assay

Cell viability in Caco-2 cells was investigated using the MTT (3-(4,5-dimethylthiazol-2-yl)-2,5-diphenyltetrazolium bromide) assay. Cells were seeded in 96-well tissue culture plates at a density 1×10^4^ cells/well. After a 24 hr treatment with Manumycin A, an MTT solution was added (10 µl/well) and incubated at 37°C for 4 hr. The medium was then removed, and the precipitated formazan crystals were dissolved in 100 µl DMSO. After shaking for 30 min, the absorbance at 570 nm was measured using a microplate ELISA reader (Biorad, Europe). The relative cell viability was calculated as follows: relative cell viability = (mean experimental absorbance/mean control absorbance)×100%. Assays were performed in triplicate on three independent experiments.

### TUNEL Assay

Cellular apoptosis in Caco-2 cells was visualized using the TUNEL (terminal deoxynucleotidyl transferase-mediated biotinylated UTP nick end-labeling) detection assay (Roche, Athens, Greece). Cells were incubated with Manumycin for 24 hr and then washed three times with PBS. Briefly, the cells were treated with Proteinase K and rinsed twice with PBS. An equilibrium buffer containing the labeled nucleotide mix and TdT enzyme was added; the mixture was allowed to incubate at 37°C for 1 hr in a 5% CO_2_ humidified chamber, and then staining was performed. Observed through a Carl Zeiss fluorescence microscope, with detection at 570 nm, normal cells are not stained whereas apoptotic cells have a strong red fluorescence.

### Statistical Analysis

Student’s t test and one-way and two-way ANOVA tests were used for statistical analyses of the data. All statistical analyses were conducted using GraphPad 5.1 (GraphPad, La Jolla, USA). Cases with P values of <0.05 or <0.01 were considered statistically significant (*P<0.05, **P<0.01, ***P<0.001).

## Results

### Interleukin and K-ras Correlation in Colorectal Cancer Stage Progression

The levels of IL-17, IL-22 and IL-23 in cancer patients and healthy controls were determined using human interleukin ELISA Immunoassay, Western Blot, and RT-PCR techniques.

### IL-17

**Figure 1:**
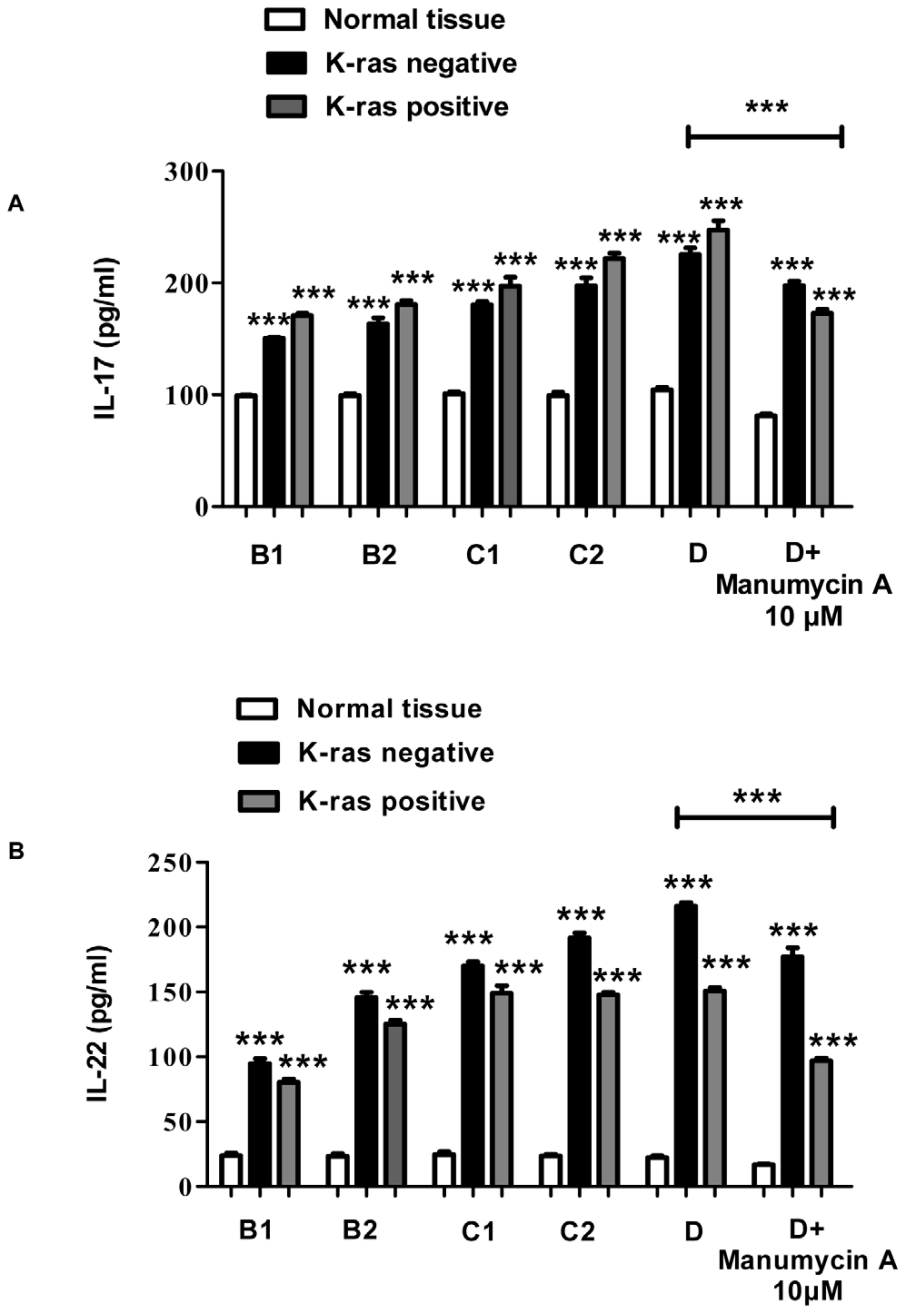
Colon cancer stage-specific correlation between interleukin levels and K-ras positive/negative patients and healthy controls. Determination of interleukin (**A**) IL-17, (**B**) IL-22, and (C) IL-23 concentrations (pg/ml) in the tumor tissues of K-ras positive, K-ras negative colon cancer patients, and control patients in the various stages of colon cancer progression. Manumycin A was applied on normal and stage D patient samples. The data are presented as mean ± SD of samples for each group involved. Large bar indicates comparison between patient stage D and stage D+Manumycin treatment samples.

**Figure 2:**
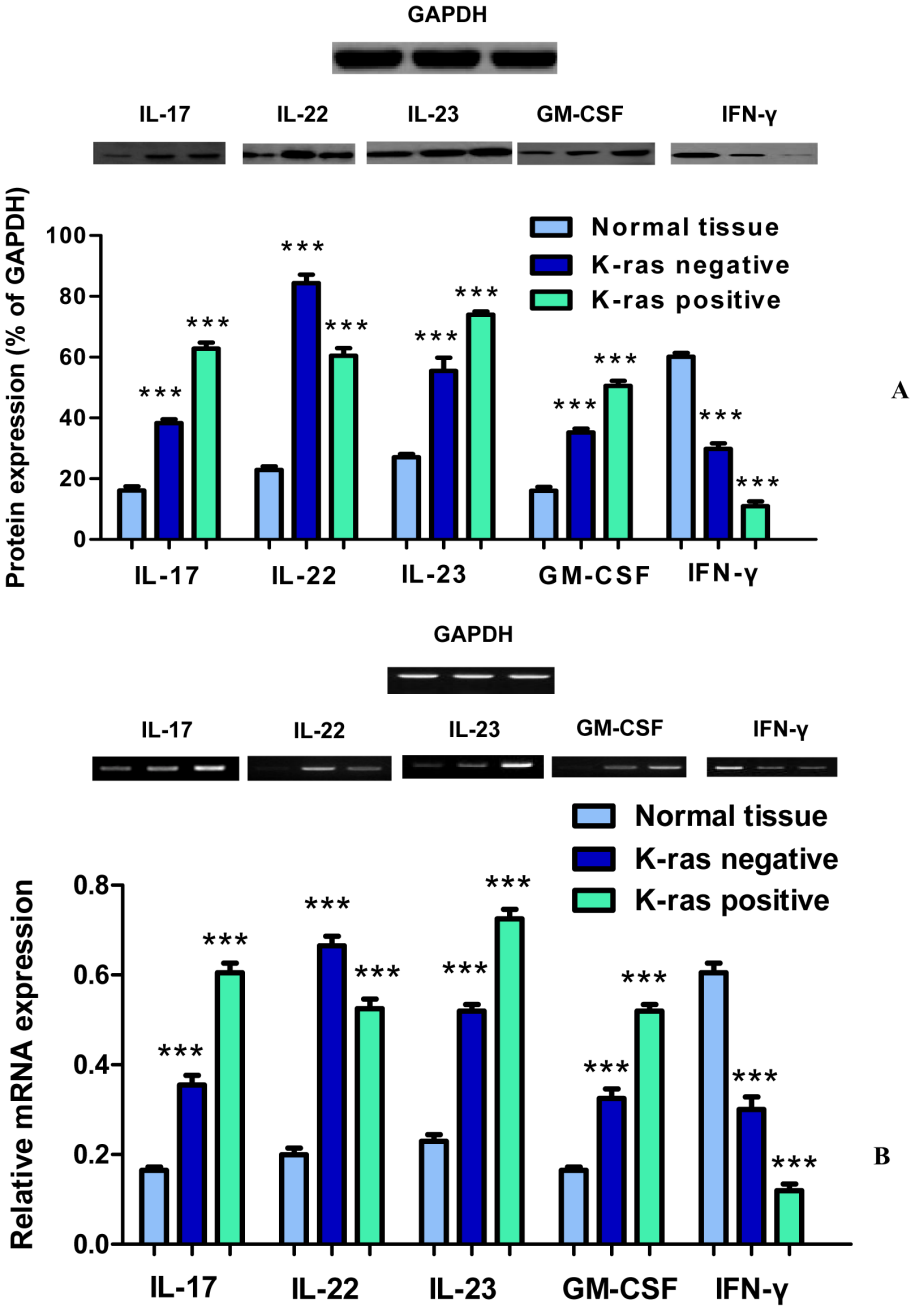
Protein and mRNA expression levels of IL-17, IL-22, IL-23, GM-CSF, and IFN-γ. (**A**) Protein expression levels of IL-17, IL-22, IL-23, GM-CSF, and IFN-γ. GAPDH was used as a loading control for Western blot analysis. Data are mean ± SD of three independent experiments. Densitometric analysis of each protein level was calculated from the average of three experiments. Each value was expressed as the ratio of the measured protein to GAPDH level (P<0.001). (**B**) mRNA expression levels of IL-17, IL-22, IL-23, GM-CSF, and IFN-γ from colon cancer tissues performed by RT-PCR analysis. 5 µg of total RNA was isolated from cancer tissues. Values represent the mean ± SD of three independent experiments.

**Figure 3:**
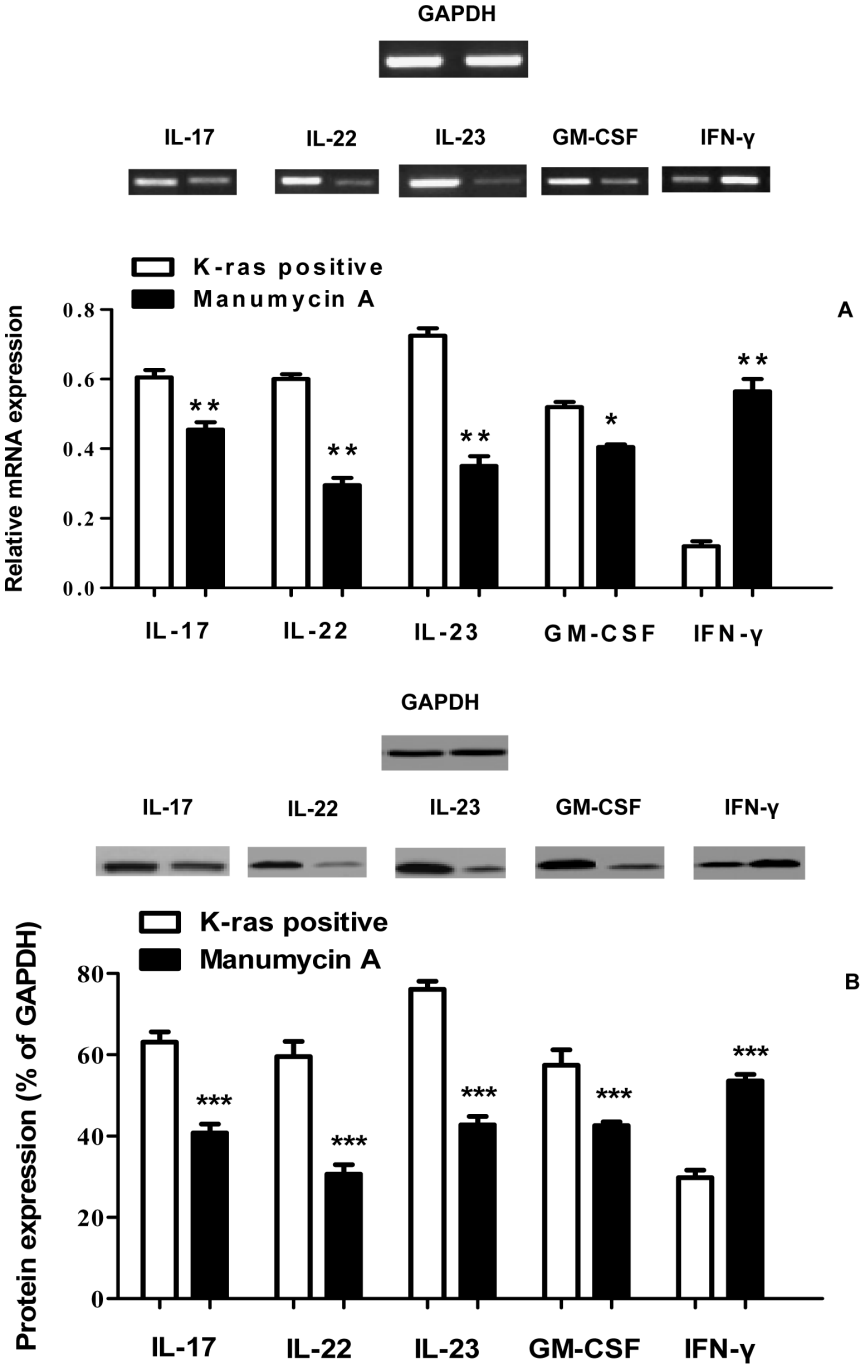
Influence of Manumycin A on protein and mRNA expression levels of IL-17/IL-22/IL-23, GM-CSF, and IFN-γ. Effect of Manumycin A treatment (10 µM) on the (**A**) mRNA expression levels of IL-17, IL-22, IL-23, GM-CSF, and IFN-γ in K-ras positive colorectal cancer patients as performed by RT-PCR analysis. Patient stage D colon cells were treated with Manumycin A (10µM) for 24 hr, before RT-PCR. 5 µg of total RNA was isolated from treated colon cells. Values represent the mean ± SD of three independent experiments. (**B**) protein expression levels of IL-17, IL-22, IL-23, GM-CSF, and IFN-γ in K-ras positive colorectal cancer patients as performed by Western Blot analysis. Patient stage D colon cells were treated with Manumycin A (10µM) for 24 hr, before protein quantification. Values represent the mean ± SD of three independent experiments.

Initially, IL-17 expression levels were measured at consecutive stages of colorectal cancer and in healthy control tissues using ELISA. The results showed that IL-17 expression levels were generally higher in K-ras positive cancer tissues (from 170 pg/ml in B1 to 247 pg/ml in D) compared to K-ras negative samples (from 150 pg/ml in B1 to 225 pg/ml in D) (P<0.05) (Figure 1A). Analysis of IL-17 protein levels along cancer stages B1-D in patients showed that they were significantly higher in comparison to the a) corresponding levels of K-ras negative patients (62% vs. 38%), and b) healthy controls (62% vs. 16%) (Figure 2A). Furthermore, the mRNA level of IL-17 was determined in K-ras positive samples. A significant rise in mRNA IL-17 levels was observed (Figure 2B). In order to examine the effect of K-ras mutation (G12V) on interleukin expression, the ras farnesyl transferase inhibitor Manumycin A was applied on normal and stage D patient samples, due to the selective overexpression of that interleukin at that particular stage. At the end of the Manumycin A treatment (10 μΜ), a significant decrease (P<0.001) in IL-17 levels was observed (Figure 1A). A similar decrease in K-ras positive stage D samples was exemplified by protein and mRNA levels (P<0.001 for protein levels, P<0.01 for mRNA levels) (Figure 3A–B). The experiments indicate the importance of mutant K-ras signaling in IL-17 expression.

### IL-22

Tissue IL-22 levels were significantly raised in the K-ras negative group compared to the patients bearing the K-ras mutation (from 94 pg/ml vs. 80 pg/ml in B1 to 216 pg/ml vs. 150 pg/ml in D) (P<0.05 for B1–C1, P<0.01 for C2–D) (Figure 1B). In addition, protein and mRNA levels of IL-22 were significantly higher in the K-ras negative group. Specifically, among these patients, protein IL-22 levels were elevated in the K-ras negative group (84% vs. 60%) (P<0.001) (Figure 2A). Moreover, IL-22 mRNA levels in the K-ras positive group were lower in comparison to the K-ras negative group (P<0.001) (Figure 2B). Addition of Manumycin A at 10 μΜ to normal and stage D patient samples caused a decrease in IL-22 levels (P<0.001 for protein levels, P<0.01 for mRNA levels) (Figure 1B, 3A–B).

### IL-23

The ELISA, mRNA, and protein analysis revealed that K-ras positive patients showed an increase in IL-23 expression compared to the K-ras negative (P<0.01 for B1, P<0.05 for B2–D) and healthy control group (P<0.001) (Figure 1C, 2A–B). To determine the correlation between K-ras presence and IL-23 expression, Manumycin A was added to normal and stage D patient samples and IL-23 protein and mRNA levels in K-ras positive patients were determined (Figure 1C, 3A–B). The results show that inhibition of K-ras causes a significant decrease in IL-23 levels (P<0.001 for protein levels, P<0.01 for mRNA levels), thereby establishing a correlation between K-ras and IL-23 levels.

### GM-CSF

**Figure 4:**
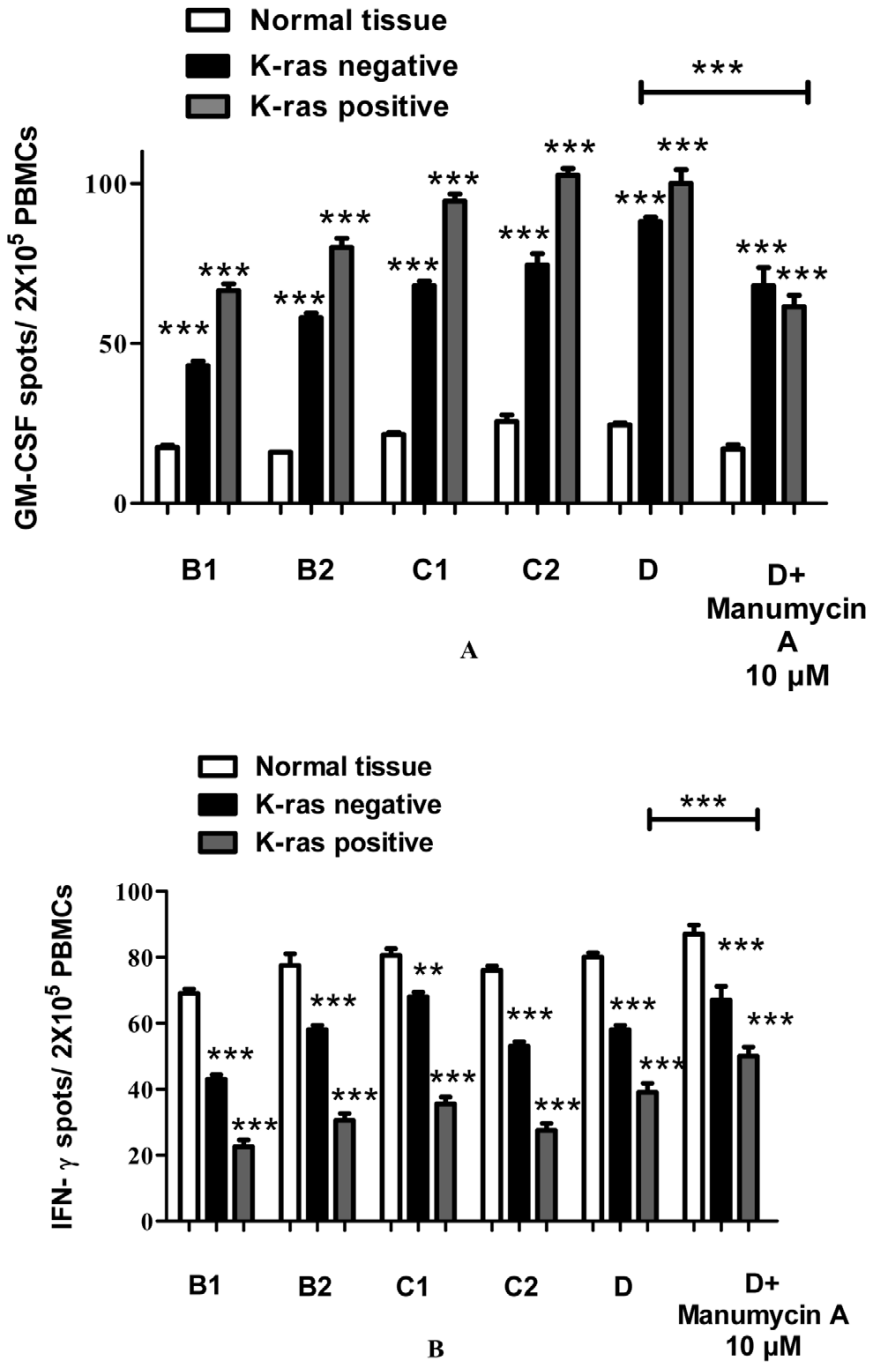
GM-CSF and IFN-γ levels of PBMCs from patients before surgery. In (**A**) GM-CSF ELISPOT of PBMCs was obtained from patients before surgery. Mononuclear cells were stimulated and were investigated for GM-CSF secretion. All experiments were carried out in triplicate. Results are presented as mean ± SD of the different groups. (**B**) IFN-γ ELISPOT of PBMCs was obtained from patients before surgery. Mononuclear cells were stimulated and were investigated for IFN-γ secretion. All experiments were carried out in triplicates. Results are presented as mean ± SD of the different groups. Large bar indicates comparison between patient stage D and stage D+Manumycin treatment samples.

To investigate potential mechanisms of GM-CSF involvement in the various stages of colon carcinogenesis, the expression of GM-CSF was monitored and quantified, by ELISPOT, Western Blot, and RT-PCR methods. For the GM-CSF ELISPOT, cells from freshly isolated PBMCs were prepared and used for GM-CSF detection. The findings show a gradual increase of GM-CSF levels during colon cancer progression, especially in stages C2 and D (P<0.001) (Figure 4A, Figure S1). Moreover, GM-CSF protein and mRNA levels were significantly higher in K-ras positive, stage D patients (P<0.001) (Figure 2A–B) compared to K-ras negative and healthy control patients, indicating an induced GM-CSF activation during colon cancer stage progression tumorigenesis (Figure 4A, Figure S1). The observed results are in agreement with recent findings indicating the importance of GM-CSF in K-ras positive colon cancer tumorigenic processes (vide infra). Manumycin A was added to normal and stage D patient samples (Figure 4A) and GM-CSF protein and mRNA levels in K-ras positive patients were determined (Figure 3A–B). The results show that inhibition of K-ras causes a significant decrease in GM-CSF mRNA and protein levels (P<0.001 for protein levels, P<0.05 for mRNA levels), thereby establishing a correlation between the two molecules.

### IFN-γ

In order to examine the effect of the K-ras mutation on IFN-γ during the progression stages of colorectal cancer, the expression level of IFN-γ was determined. As shown (Figure 4B, 2A–B, Figure S1), IFN-γ is down-regulated in colon cancer patients compared to healthy controls. In addition, there is a significant decrease in IFN-γ protein and mRNA levels between K-ras negative and K-ras positive individuals (P<0.001), thereby pointing out the effect of K-ras signaling on IFN-γ down-regulation. Furthermore, treatment with Manumycin A caused a rise in IFN-γ expression levels, as shown by Western Blot and RT-PCR experiments (P<0.001 for protein levels, P<0.01 for mRNA levels) (Figure 3A–B). The results suggest a K-ras feedback signaling mechanism on IFN-γ, which plays an important role in colon carcinogenesis.

### Manumycin A Suppression of the Viability and Cell Growth of Caco-2 Cells

**Figure 5:**
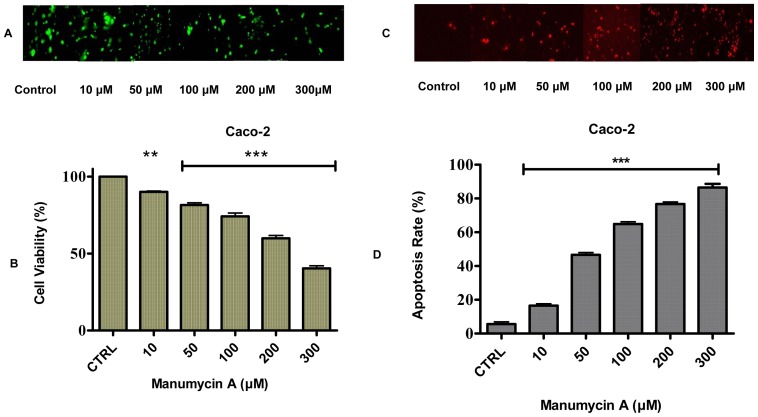
Effect of Manumycin A on Caco-2 cell viability and apoptosis. (**A**) MTT assay showing the effect of Manumycin A on cancer cell viability in Caco-2 cells. Cells were treated with different concentrations of Manumycin A for 24 hr. Cell viability was measured with an ELISA plate reader at 570 nm. Image magnification ×200. (**B**) The results indicate that Manumycin A inhibits the viability of human colon adenocarcinoma Caco-2 cells in a dose-dependent fashion. Values represent the mean ± SD of three independent experiments. Manumycin A induces apoptosis in (**C**) Caco-2 human colon adenocarcinoma cancer cells. Caco-2 cell apoptosis was detected using the TUNEL detection assay from Roche. Manumycin A concentrations: 10, 50, 100, 200, and 300 μΜ. The cancerous cell line was exposed to Manumycin A at different concentrations for 24 hr and stained. Red apoptotic cells were viewed using a Carl Zeiss (Zeiss Europe) fluorescent microscope. Image magnification ×200. (**D**) The graphs represent the quantitative results of apoptosis. Data are mean ± SD of three independent experiments.

The observed results show for the first time that ras inhibitor Manumycin A reduces cell viability in Caco-2 colon cancer cells. The effect of this specific form of inhibitor on the viability of colon cancer cells was examined after incubation in a medium containing Manumycin A with concentrations in the range 10–300 µM for 24 hr. Cells incubated in the medium in the absence of Manumycin A served as controls. Employment of the MTT assay revealed that inhibition of cell viability in all Caco-2 colon adenocarcinoma cancer cells tested occurred in a dose-dependent fashion (Figure 5A–B). In detail, cell viability decreased significantly from 90% (at 10 μΜ) to 40% (at 300 μΜ) (P<0.001), compared to the control. The observed patterns of inhibition as a function of Manumycin A concentration reveal the antitumor properties of ras inhibitors in K-ras induced colon cancer signaling.

### Manumycin A-induced Dose-dependent Apoptosis

TUNEL apoptosis detection staining assays were employed to ascertain the apoptotic effect of Manumycin A on Caco-2 cells following a 24 hr treatment (Figure 5C–D). The substantial apoptotic changes observed in the Manumycin A-treated Caco-2 cells for 24 hr were studied and photographed with a Carl Zeiss confocal microscope. Apoptotic cells became round with red fluorescence. As shown in Figure 5D, exposure to Manumycin A increased the apoptotic rate in colon adenocarcinoma Caco-2 cells. In particular, early apoptotic cells rose significantly from 16% (at 10 μΜ) to 86% (at 300 μΜ) (P<0.001), compared to the control. The effect of Manumycin A on the cancer cell line reveals that the rise in the apoptotic rate is dose-dependent and the Caco-2 cells are sensitive to the ras inhibitor treatment even at low concentrations. The results suggest a Manumycin A-induced apoptosis in colon cancer cells and a key role of the specific inhibitor in colon antitumor activity.

## Discussion

### Crosstalk in Interleukin – K-ras Correlations

Mutations in the K-ras oncogene are often associated with distinct clinical and pathological characteristics [16], [17]. Prominent among such mutations is the G12V one, which characterizes the K-ras oncogene employed in this work. Although several studies have reported interleukin over-expression in K-ras positive cancer patients [18], [19], this is the first time that IL-17, IL-22, and IL-23 are associated with K-ras-linked cancer stage-specific over-expression. Despite the fact that the association mechanism between interleukins and K-ras remains unknown, activation of the RAS-ERK-MAPK pathway has been shown to trigger various immunological responses [20]. In this regard, a latest study revealed that oncogenic K-ras-induced IL-8 over-expression a) promotes cell growth and migration, and b) contributes to aggressive phenotypes of non-small lung cancer cells [21]. Likewise, recent work on a similar subject investigated mechanisms responsible for tumor-elicited inflammation in a mouse model for colorectal tumorigenesis, which like human colorectal cancer exhibits up-regulation of IL-17 and IL-23 [22]. Furthermore, mutated ras can induce expression of IL-1b, which can then serve as an autocrine growth factor for leukemic cells [23]. For that reason, molecules designed to inhibit ras function and/or ras expression, like FTase inhibitors, are being used in clinical trials for different types of tumors with very promising results in potential cancer therapeutics [24], [25].

### GM-CSF – K-ras Interplay

GM-CSF is a well-known hematopoietic and inflammatory cytokine produced by macrophages, T cells, mast cells, NK cells, endothelial cells and fibroblasts[26]. It’s involved in the activation of several intracellular signaling pathways including the JAK/STAT, RAS/ERK, and PI3K/AKT signaling pathways [27], [28]. Furthermore, it can induce cell survival through activation of NF-kB and expression of the anti-apoptotic protein Bcl-2 [29]. The present study showed that colorectal cancer patients and in particular K-ras positive individuals express GM-CSF at higher levels compared to their normal counterparts. The results indicate that the cytoprotective effects of GM-CSF are mediated through the RAS/MAPK signaling pathway, as shown by the K-ras inhibitor in the RT-PCR and the Western Blot experiments. Our work is in agreement with recent findings reporting that oncogenic K-ras induces GM-CSF production, in turn promoting the development of pancreatic neoplasia [30]. Additionally, it’s been reported recently that the endogenous oncogenic N-ras G12D mutation promotes aberrant GM-CSF chronic myelomonocytic leukemia in mice [31]. Furthermore, when patients with pancreatic adenocarcinoma were vaccinated with synthetic mutant ras peptides, in combination with GM-CSF, showed promising results [32]. The above findings reveal that aberrant GM-CSF signaling drives inappropriate cell growth and survival during disease initiation, progression, and malignant transformation and as a result GM-CSF binds to its receptor to promote cell survival, proliferation, and differentiation.

### IFN-γ – K-ras Association

In contrast to the above observations on GM-CSF, a significant reduction in the level of IFN-γ was observed in all cancer patients in comparison to the control. This can be explained by the fact that IFN-γ is a cytokine bearing both immunostimulatory and immunomodulatory effects against carcinogenesis and tumor growth by inhibiting cell growth and inducing cell death in cancer cells [33], [34]. In addition, IFN-γ also has important immunoregulatory functions, including activation of macrophages, induction of antiproliferative effects on transformed cells, and can potentiate antiviral and antitumor effects of the type brought about by interferons such as IFN-α and IFN-β1a [35]. Aberrant IFN-γ expression is associated with a number of autoinflammatory and autoimmune diseases. To this end, interferon IFN-γ can act as a pro-inflammatory cytokine, affecting the regulation of cell proliferation. Its administration in clinical trials has been shown to have a positive influence on patient survival outcome in both bladder cancer and lung cancer [36], [37]. Evidence for the beneficial effects of IFN-γ on cancer has been provided by several studies in the last decade [38], [39]. Another mechanism of inhibition of tumor cell proliferation might be the ability of IFN-γ to down-regulate the expression of many proto-oncogenes such as HER-2/neu in malignant cells [40]. As a result, IFN-γ is used worldwide for the treatment of more than 20 types of cancers including some hematological malignancies (chronic myeloid leukemia, B- and T-cell lymphoma) and certain solid tumors such as melanoma and Kaposi’s sarcoma [41], [42], [43]. In a recent publication, a combination of IFN-*α* and antisense K-ras mRNA emerged as a promising gene therapy strategy against pancreatic cancer [44].

### Inflammation – Immune Responses in Colorectal Cancer

The molecular mechanisms by which inflammation promotes cancer development are still being uncovered and could differ among the various stages of colorectal cancer progression [45], [46]. Nevertheless, the link between these mechanisms lies in the common genetic and signaling pathways, like Wnt, β-catenin, K-ras, p53, transforming growth factor TGF-β, tumor necrosis factor TNF-a, and the DNA mismatch repair (MMR) proteins, which are altered in sporadic colorectal cancer through specific mutations in these molecules [47]. Over the past years, it has been reported that chronic inflammation, leading to early stages of colorectal cancer, is characterized by the production of pro-inflammatory cytokines, which can induce a) mutations in oncogenes and tumor suppressor genes (APC, p53, K-ras), and b) genomic instability through a variety of mechanisms [48]. Persistent inflammation facilitates tumor promotion by activating proliferation and anti-apoptotic properties of premalignant cells, as well as tumor progression and metastasis. Furthermore, a large proportion of colorectal tumors and human colorectal cell lines exhibit constitutive activation of transcription factors, which are essential components of multiple inflammatory pathways such as nuclear factor-kB (NF-kB) and signal transducer and activator of transcription 3 (STAT3). In particular, the constitutively active NF-kB is associated with the “damaging” features of malignant cells, such as anti-apoptotic effects, cell growth, and metastasis. These mutated genes provide a growth advantage that drives tumor progression as successive clonal outgrowths are generated, ultimately forming carcinoma.

### The use of K-ras and other Inhibitors in the Treatment of Colorectal Cancer

**Figure 6:**
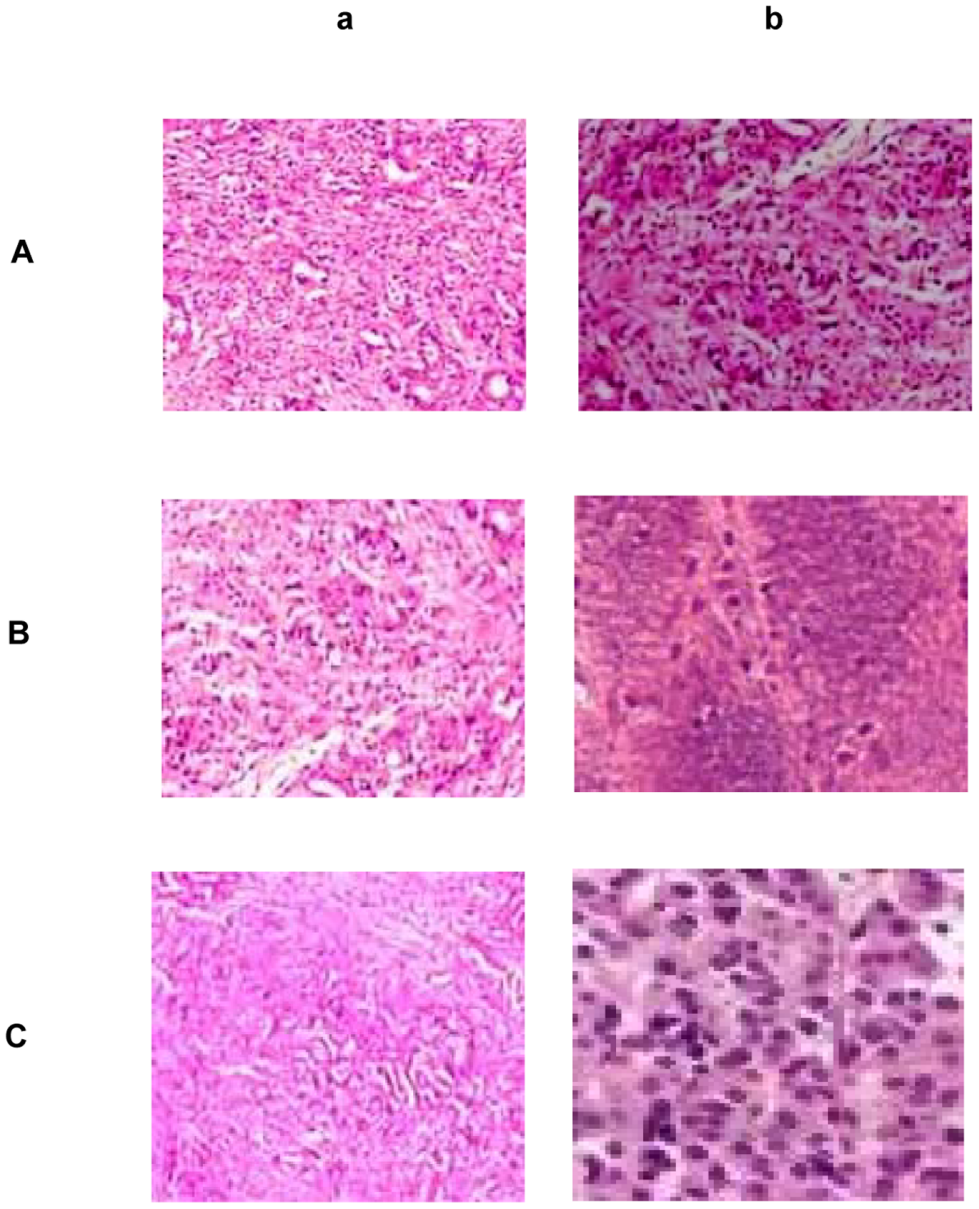
Immunohistochemical analysis of IL-17, IL-22, and IL-23 in human colon biopsies. Immunohistochemical staining of (**A**) IL-17, (**B**) IL-22, and (**C**) IL-23 in colon biopsies from patients with colorectal cancer. Paraffined sections of human colon biopsies were obtained from patients with colorectal cancer (**b**), and non-cancerous tissue (**a**). Human IL-17, IL-22, and IL-23, were detected using anti-human monoclonal antibodies from Santa Cruz. Staining with mouse IgG1 isotype was used as the negative control. Stained tumor cells are shown at a final magnification of ×400.

The findings of this work indicate that mutant K-ras in various stages of colorectal cancer development is associated with elevated levels of interleukin expression in colorectal tissue. These observations are evident in precursor lesions within the colon epithelial microenvironment as shown by immunohistochemistry results (Figure 6). Mutations in K-ras in colorectal cancer are also unique in that they typically involve codon 12, but may also rarely involve codons 13 or 61 [6]. These mutations render K-ras resistant to GAP and as a result lead to constitutive activation of downstream pathways, resulting in altered regulation of cellular proliferation. Hence, K-ras inhibitors of downstream signaling pathway molecules, such as RAF/MAPK or PI3K, emerge as important tools in combating inflammation-linked tumorigenesis. In the present work, employment of ras inhibitor Manumycin A results in lowering GM-CSF and raising IFN-γ levels, thereby reducing inflammatory-driven tumorigenic responses. Similar arguments can be made about other inhibitors to key molecular targets such as BRAF or EGFR [49], PARP [50], and COX-2 [51], thereby emphasizing the importance of such molecules in gene-specific target cancer therapy. Recent clinical trials include both farnesyl transferase inhibitors and antisense oligonucleotides in an effort to prevent metastasis of colon adenocarcinoma cells. Given the high frequency of oncogenic K-ras in colorectal adenocarcinoma, particularly in one codon, therapeutic approaches that are codon-specific might be of greater benefit. Although an antisense oligonucleotide against K-ras has been developed [52], [53], no such molecule has yet entered clinical trials. To this end, such stage specific antisense oligonucleotides targeting mutant K-ras signaling in colorectal cancer emerge as promising therapeutic targets.

## Conclusions

Collectively, the present study demonstrates the critical role that specific interleukins play in inflammation-driven tumorigenesis and provides new insights into the aberrant cytokine signaling in oncogenic K-ras associated colorectal carcinogenesis. Given the role of ras signaling in cancer progression and metastasis, this study can serve as a platform for the identification and validation of synergistic biomarkers with oncogenic K-ras toward development of therapeutically efficacious molecular agents targeting these mutations. The findings a) project important implications for further design of novel therapies for patients with colorectal adenocarcinoma, and b) shed light on molecular mechanisms involving crosstalk between tumor cells and the immune system by targeting exclusive members of the cytokine superfamily that regulate tumor growth and surveillance.

## Supporting Information

Figure S1 **GM-CSF and IFN-γ levels of PBMCs from patients before surgery.** In **(A)** GM-CSF ELISPOT of PBMCs was obtained from patients before surgery. Mononuclear cells were stimulated and were investigated for GM-CSF secretion. All experiments were carried out in triplicate. Results are presented as mean ± SD of the different groups. **(B)** IFN-γ ELISPOT of PBMCs was obtained from patients before surgery. Mononuclear cells were stimulated and were investigated for IFN-γ secretion. All experiments were carried out in triplicates. Results are presented as mean ± SD of the different groups. Large bar indicates comparison between patient stage D and stage D+Manumycin treatment samples.
(TIFF)

Table S1 Clinical characteristics of colorectal cancer patients.
(TIF)

Table S2 List of primers used in the RT-PCR gene expression studies.
(TIF)
