# Supplementary figures and images for: Differential Expression of IL-17, 22 and 23 in the Progression of Colorectal Cancer in Patients with K-ras Mutation: Ras Signal Inhibition and Crosstalk with GM-CSF and IFN-γ
Source: PLoS One. 2013 Sep 6;8(9):e73616. doi: 10.1371/journal.pone.0073616 (PMC3765247; doi:10.1371/journal.pone.0073616)

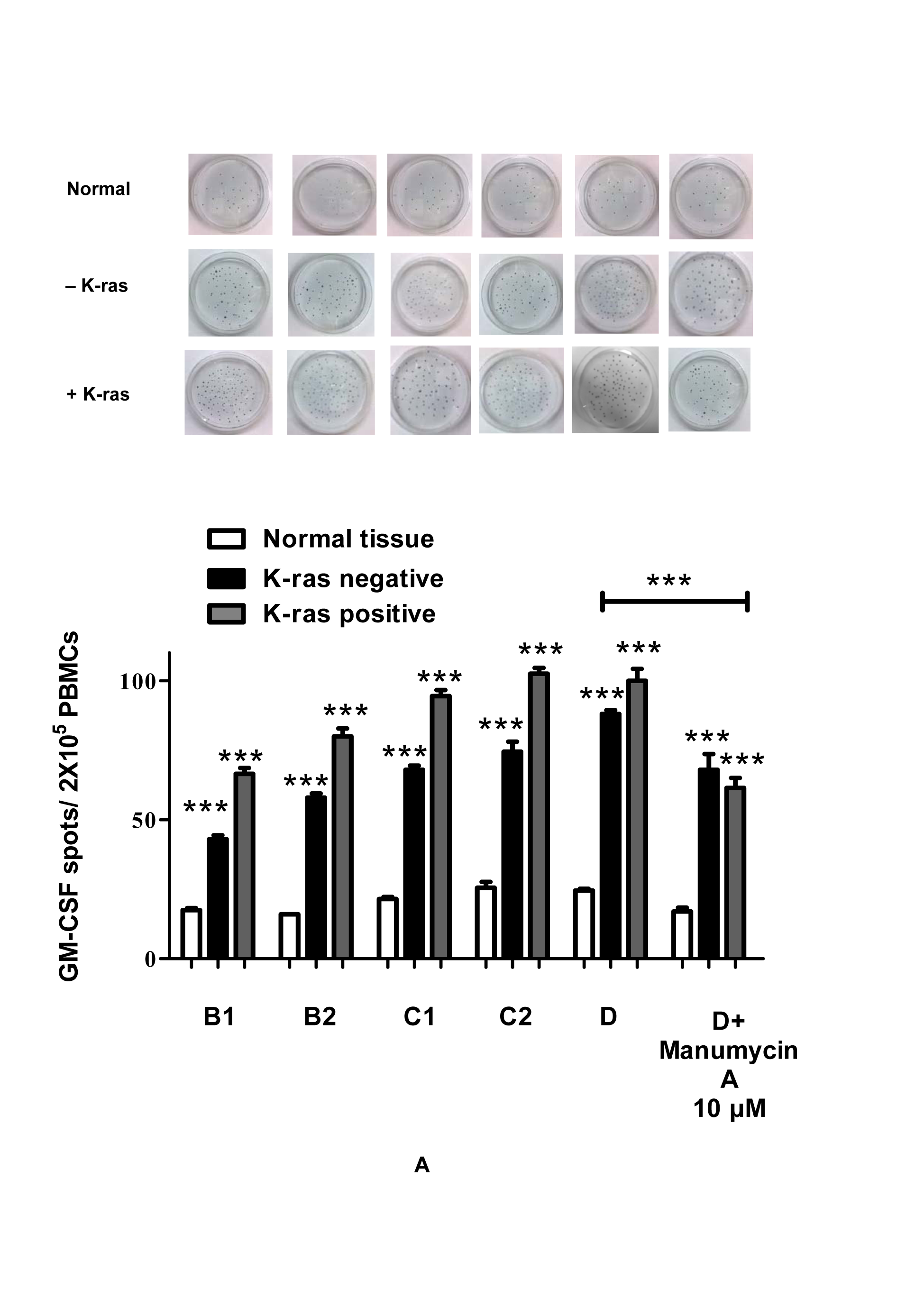

Supplement: Figure S1 — GM-CSF and IFN-γ levels of PBMCs from patients before surgery. In (A) GM-CSF ELISPOT of PBMCs was obtained from patients before surgery. Mononuclear cells were stimulated and were investigated for GM-CSF secretion. All experiments were carried out in triplicate. Results are presented as mean ± SD of the different groups. (B) IFN-γ ELISPOT of PBMCs was obtained from patients before surgery. Mononuclear cells were stimulated and were investigated for IFN-γ secretion. All experiments were carried out in triplicates. Results are presented as mean ± SD of the different groups. Large bar indicates comparison between patient stage D and stage D+Manumycin treatment samples. (TIFF) [file pone.0073616.s001.tiff]

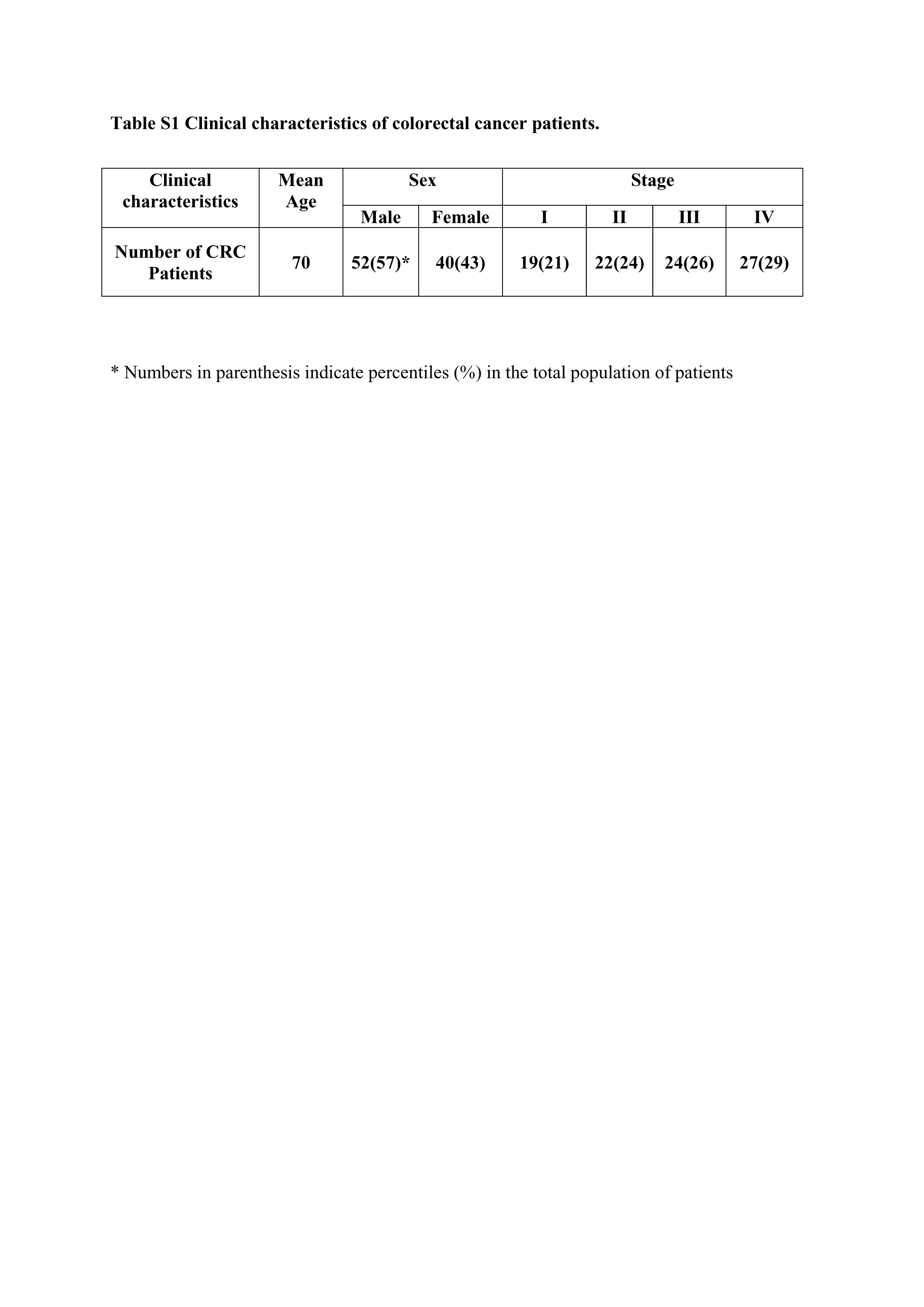

Supplement: Table S1 — Clinical characteristics of colorectal cancer patients. (TIF) [file pone.0073616.s002.tif]

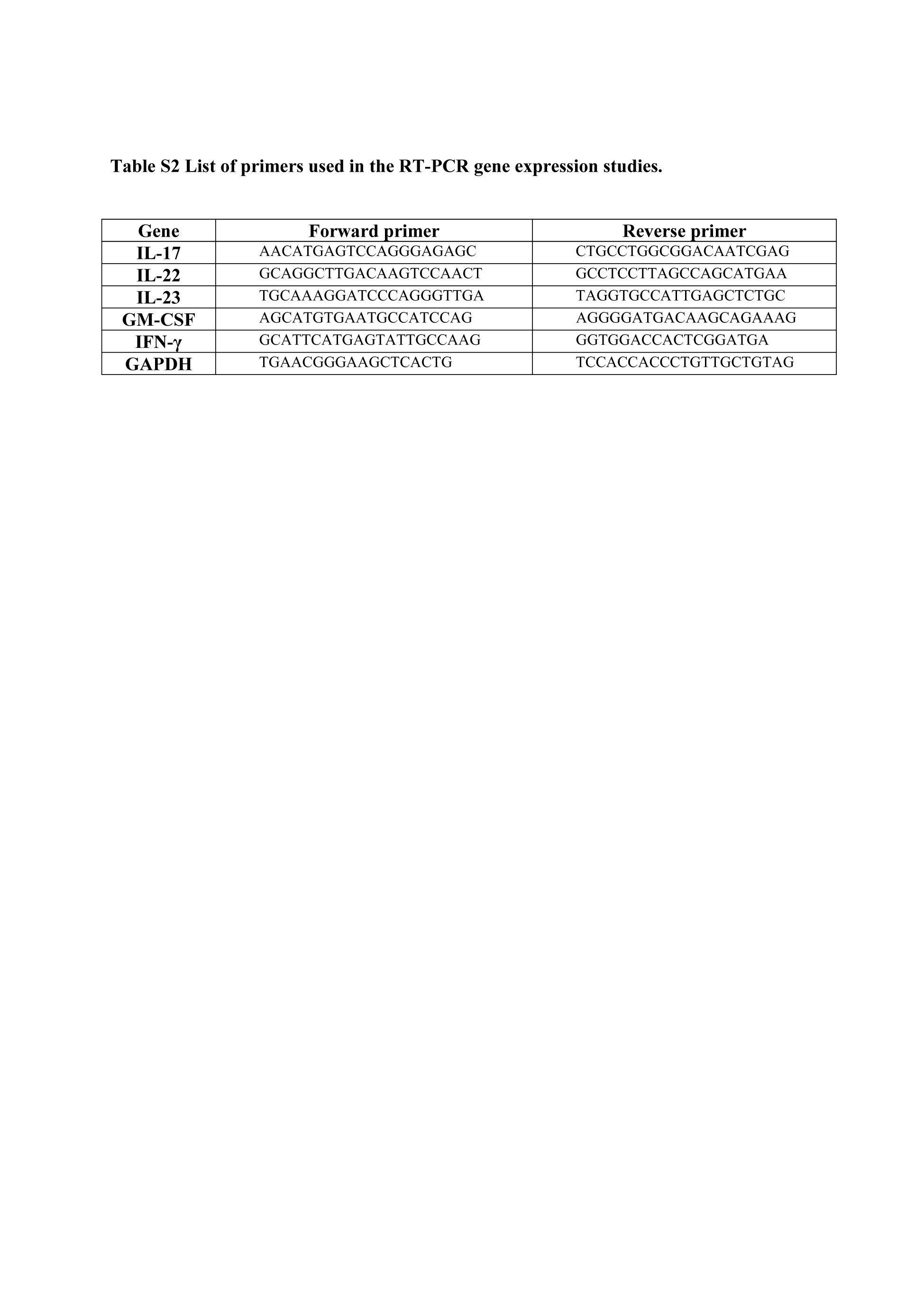

Supplement: Table S2 — List of primers used in the RT-PCR gene expression studies. (TIF) [file pone.0073616.s003.tif]
